# A Facile One-Pot Approach to the Synthesis of Gd-Eu Based Metal-Organic Frameworks and Applications to Sensing of Fe^3+^ and Cr_2_O_7_^2−^ Ions

**DOI:** 10.3390/s21051679

**Published:** 2021-03-01

**Authors:** Roberta Puglisi, Anna L. Pellegrino, Roberto Fiorenza, Salvatore Scirè, Graziella Malandrino

**Affiliations:** 1 Dipartimento di Scienze Chimiche, Università degli Studi di Catania, Viale Andrea Doria 6, I-95125 Catania, Italy; robertapuglisi90@gmail.com (R.P.); rfiorenza@unict.it (R.F.); sscire@unict.it (S.S.); 2 Dipartimento di Scienze Chimiche, Università degli Studi di Catania, INSTM UdR Catania, Viale Andrea Doria 6, I-95125 Catania, Italy; annalucia.pellegrino@unict.it

**Keywords:** Gd,Eu-MOF, ion sensing, X-ray powder pattern, ditopic linkers, porosity, surface area

## Abstract

Gadolinium metal-organic frameworks (Gd-MOFs) and Eu-doped Gd-MOFs have been synthesized through a one-pot green approach using commercially available reagents. The 1,4-benzenedicarboxylic acid (H_2_-BDC) and 2,6-naphthalenedicarboxylic acid (H_2_-NDC) were chosen as ditopic organic linkers to build the 3D structure of the network. The Gd-MOFs were characterized using powder X-ray diffraction (XRD), FT-IR spectroscopy, field emission scanning electron microscopy (FE-SEM) and N_2_ adsorption–desorption analysis. The Gd-MOF structures were attributed comparing the XRD patterns, supported by the FT-IR spectra, with data reported in the literature for Ln-MOFs of similar lanthanide ionic radius. FE-SEM characterization points to the effect of the duration of the synthesis to a more crystalline and organized structure, with grain dimensions increasing upon increasing reaction time. The total surface area of the MOFs has been determined from the application of the Brunauer–Emmett–Teller method. The study allowed us to correlate the processing conditions and ditopic linker dimension to the network surface area. Both Gd-MOF and Eu-doped Gd-MOF have been tested for sensing of the inorganic ions such as Fe^3+^ and Cr_2_O_7_^2−^.

## 1. Introduction

During the last decades, metal-organic frameworks (MOF) have shown interesting properties which lead to important applications in several fields of material science such as energy transfer and light-harvesting [1,2], gas storage [3,4,5], adsorption of hydrocarbons [6,7], sensing [8,9], catalysis [10,11,12] and ferroelectrics [13]. In addition, MOFs represent platforms for functional materials [14] and recently they have been found appealing for photovoltaic applications [15]. The advantages of these materials are the possibility of tuning their properties by easily altering the structure, e.g., by changing the metallic center, the organic linkers and the synthetic operating conditions such as duration, temperature and pressure. In regard to the linker, a variety of multitopic spacer have been applied and a plethora of MOF structures have been obtained [16]. These materials are composed of organic secondary subunits and/or metal containing ones. The shape of these clusters can be assimilated to specific geometric structures, such as triangular ones, tetrahedral or octahedral in periodically organized systems. For example, among the most studied MOFs of transition metals, there are the MOF-5 and IRMOF-5, consisting of Zn_4_O-cluster sub-units with 1,4-benzendicarboxylic acid and Zn_4_O-clusters with 2,6-naphthalendicarboxylic acid [17,18,19].

In regard to the metal center, great attention has been devoted to transition metals, due to their appealing properties tunable by molecular design [20,21,22]. Recently, the attention has moved to lanthanides (Ln) due to their fascinating properties ranging from metal ion sensing to ratiometric thermometry, from magnetic to luminescence, from gas and dyes adsorption to catalysis. A comprehensive view of the various application fields of Ln-MOF can be found in the reviews [23,24,25] and references therein.

The use of these elements allowed the development of specific properties and in particular Eu-based MOFs have recently attracted great attention, due to their applications to sensing a great variety of species. Many studies have been carried out on the detection of pollutants, such as some inorganic ions in water, because these chemical species pose serious problems for water contamination. In particular, the detection process of inorganic cations and toxic anions is primarily based on quenching of fluorescence phenomena, arising from europium ions sensitized by the MOF ligand through the “antenna effect” [26,27,28,29,30,31]. The Eu-based MOFs have also been applied to detect antibiotics, which are widely used for the treatment of bacterial infections and are excreted generating major organic pollutants in water [28,32]. The Eu-based MOFs have also been shown to be highly sensitive for nitroaromatic explosives [32,33], and have been tested for detection of amino acids [34] and adsorption of dyes [35,36].

Up to date, syntheses of Ln-MOFs have been essentially carried out through microwave, electrochemical or mechanochemical processes and solvothermal route [37,38,39], where high pressure, organic solvents and, in some cases, very long reaction times are required. Recently, the need to develop an up-scalable, green synthetic process, of great interest in terms of industrial requirements, has been the focus of a review addressing the green synthesis of transition metal MOFs [40].

In this context, a green, one-step route under mild conditions is highly desirable for the synthesis of Ln-MOFs in large scale in a controlled and reproducible fashion. Furthermore, an added value for these systems would be their stability in water, which would allow their applications for highly selective and sensitive luminescence sensing for ions and antibiotics in aqueous solutions.

In this study, we report a one-pot approach under mild conditions of Gd-MOF networks and 20% Eu-doped Gd-MOF based on the ditopic 1,4-benzenedicarboxylic acid (H_2_-BDC) or 2,6-naphthalenedicarboxylic acid (H_2_-NDC) linkers, yielding respectively the [Gd_2_(BDC)_3_(H_2_O)_4_] and [Gd_1.6_Eu_0.4_(BDC)_3_(H_2_O)_4_], from now on Gd-BDC and Gd,Eu-BDC, and the [Gd_2_(NDC)_3_(H_2_O)]·2(H_2_O), Gd-NDC networks. The X-ray diffraction (XRD) and field emission scanning electron microscopy (FE-SEM) allowed to study the effect of the reaction time on the structural and morphological properties, while the N_2_ physisorption, using the Brunauer–Emmett–Teller (BET) method [41], allowed to rationalize the textural properties (surface area) of the system in relation to the ditopic linker and the reaction time. Finally, based on the luminescence study, the Gd-BDC and Gd,Eu-BDC MOFs have been applied to the sensing of Fe^3+^ and Cr_2_O_7_^2−^ inorganic ions in aqueous solutions.

## 2. Materials and Methods

### 2.1. Materials

Gadolinium(III) and europium(III) acetates tetrahydrate were purchased from STREM Chemicals, while the 1,4-benzenedicarboxylic acid and the 2,6-naphthalene-dicarboxylic acid were purchased from Sigma-Aldrich. Deionized water was used as solvent.

### 2.2. Synthesis of [Gd_2_(BDC)_3_(H_2_O)_4_]

An amount of 1.2138 g (2.99 mmol) of Gd(CH_3_COO)_3_·4H_2_O was placed in a grounded-neck glass balloon. Then, 0.358 g of NaOH and 30 mL of deionized H_2_O were added and the whole system was placed over a magnetic stirrer plate. It was possible to appreciate the immediate precipitation of Gd-hydroxide. The temperature was then raised to 100 °C and after about 5 min 0.7443 g (4.48 mmol) of H_2_-BDC were added. A white precipitate formed immediately and was left stirring under reflux for 1, 6, 12 and 24 h, respectively. After the chosen reaction time, the MOF precipitate was recovered by filtration, while the Na-acetate by-product, soluble in water, was easily removed in the liquid waste. The product was washed with H_2_O and dried at room temperature. Yields were about 80–84%.

### 2.3. Synthesis of [Gd_1.6_Eu_0.4_(BDC)_3_(H_2_O)_4_]

The Gd,Eu-BDC compound was synthesized following a procedure similar to that described for the analogous Gd-BDC compound from Gd(CH_3_COO)_3_·4H_2_O (0.970 g, 2.39 mmol), Eu(CH_3_COO)_3_·4H_2_O (0.240 g, 0.6 mmol), NaOH (0.358 g, 8.96 mmol) and H_2_-BDC (0.7443 g, 4.48 mmol). Yields were about 84–86%.

### 2.4. Synthesis of [Gd_2_(NDC)_3_(H_2_O)]·2(H_2_O)

The Gd-NDC compound was synthesized following the procedure described for the analogous Gd-BDC compound from Gd(CH_3_COO)_3_·4H_2_O (1.2137 g, 2.99 mmol), NaOH (0.360 g, 8.96 mmol) and H_2_-NDC (0.9685 g, 4.48 mmol). Yields were about 82–85%.

### 2.5. Gd-MOF Characterization

Fourier transform infrared (FT-IR) spectra were recorded on an FT-IR-430 JASCO spectrometer. FT-IR spectra were recorded on nujol mull of the samples placed between KBr plates. XRD patterns were recorded on a Smartlab Rigaku diffractometer, equipped with a rotating anode of Cu Kα radiation operating at 45 kV and 200 mA. A 0.02° increment step was used for the acquisition. In order to characterize the synthesized Gd-MOF through FE-SEM images, the samples were stuck on Al stub by means of graphite double-sided adhesives. The samples were sputtered with Au in order to avoid excessive charging effects due to their non-conductive nature. FE-SEM images were taken using a ZEISS SUPRA 55 VP field emission microscope. To reduce the charging effect and to avoid sputtering with Au a low voltage of 3 kV was used. Energy dispersive X-Ray analyses were carried out using an Oxford X-ACTA detector with a beam energy of 15 keV. The N_2_ adsorption-desorption experiments were carried out in a Sorptomatic 1990 Micropore configuration (Thermo Quest). Before tests, the samples were degassed at 120 °C at 10^−3^ Torr. The physisorption experiments were performed at liquid nitrogen boiling temperature (77 K). Surface area was evaluated with the help of a fully computerized unit attached to the Micropore unit system using the BET equation [41].

For each experiment of sensing, 2 mg of [Gd_1.6_Eu_0.4_ (BDC)_3_(H_2_O)_4_] was dispersed in 2 mL of water. After that, various rates of FeCl_3_ 1 × 10^−2^ M or K_2_Cr_2_O_7_ 1 × 10^−2^ M contaminant solutions were added in the [Gd_1.6_Eu_0.4_ (BDC)_3_(H_2_O)_4_] dispersion. The mixtures were used after 5 min of sonication treatment after each addition for luminescence measurements. Sequential Fe^3+^ detection tests were carried out using the same Gd,Eu-BDC MOF powder. After each detection test, the suspension was centrifuged for 10 min in order to remove the contaminant solution and was washed with H_2_O before the next sensing test. Photoluminescence spectra were collected at room temperature using a JASCO FP-8300 spectrofluorimeter at λ excitation of 265 or 285 nm.

## 3. Results and Discussion

### 3.1. Synthesis of Gd-MOFs with Ditopic Linkers

Two different types of Gd-MOFs have been synthesized using ditopic linkers such as 1,4-benzenedicarboxylic acid (H_2_-BDC) and the 2,6-naphthalene-dicarboxylic acid (H_2_-NDC), whose carboxylated functions are capable to fit the coordination prerequisite of the metallic center and generate the hybrid polymeric structure. The Gd-BDC MOF has been also doped with Eu ion.

The syntheses have been carried out using commercially available reagents, under mild conditions of pressure and temperature. In addition, the use of water as a solvent characterizes this synthetic approach as an absolutely green route. All the syntheses were carried out under reflux conditions, while the reaction times were varied from 1 h to 24 h to correlate the structural/morphological properties to the synthetic conditions.

The synthesis of the Gd-MOFs with H_2_-BDC and H_2_-NDC acids is described in Equations (1) and (2), respectively:

2Gd(CH_3_COO)_3_·4H_2_O + 3H_2_-BDC + 6NaOH → [Gd_2_(BDC)_3_(H_2_O)_4_] + 6CH_3_COONa + 10H_2_O (1)

2Gd(CH_3_COO)_3_·4H_2_O + 3H_2_-NDC + 6NaOH → [Gd_2_(NDC)_3_(H_2_O)]·2(H_2_O) + 6CH_3_COONa +10H_2_O (2)

The present synthetic strategy represents a simple, one-pot route to the formation of Ln-MOF with respect to the time demanding solvothermal processes. It also presents some advantages with respect to the previously reported syntheses carried out in water [42,43], which require three different steps, namely, (i) the synthesis of the lanthanide chlorides through reaction of the respective oxides with hydrochloric acid; (ii) synthesis of the sodium salt of the carboxylic acid; (iii) synthesis of the Gd-MOF from the formerly synthesized reagents.

### 3.2. Structural, Morphological and Compositional Properties of [Gd_2_(BDC)_3_(H_2_O)_4_]

**Figure 1:**
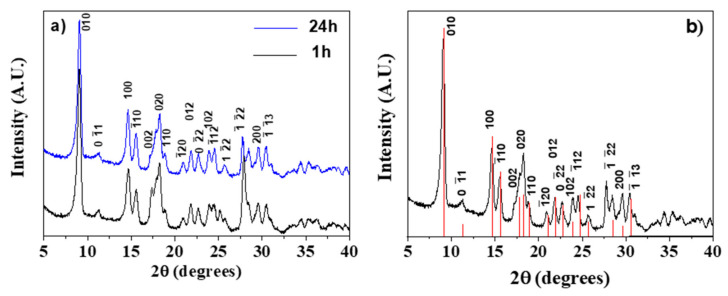
(**a**) Comparison of the X-ray powder patterns of the [Gd_2_(BDC)_3_(H_2_O)_4_] complex synthesized for 1 and 24 h; (**b**) X-ray powder pattern of the [Gd_2_(BDC)_3_(H_2_O)_4_] complex synthesized for 24 h assigned considering the powder pattern of the [Tb_2_(BDC)_3_(H_2_O)_4_] isomorph (red lines in the figure) derived from ref. [44] using the Mercury code [45].

All the syntheses, whatever the duration time, yielded crystalline samples. In Figure 1a, a comparison between the X-ray diffraction (XRD) patterns of Gd-BDC samples obtained at 1 and 24 h is reported. The reaction time, being the reaction temperature 100 °C for all the syntheses, has a slight effect on crystallinity. The diffraction pattern has been interpreted by comparison with literature data. In fact, it has been reported that M_2_(BDC)_3_(H_2_O)_4_ samples, where M is a small ionic radius lanthanide, have isomorphic structures, while the patterns are slightly different if M is a lanthanide with a larger ionic radius, for example La [43].

In Figure 1b, the XRD pattern of the Gd-BDC sample, obtained from the 24 h synthesis, is reported and compared with the theoretical data of the [Tb_2_(BDC)_3_(H_2_O)_4_] isomorph [44]. It is important to highlight that the relative intensities of the studied sample are comparable to those of the powder pattern of the [Tb_2_(BDC)_3_(H_2_O)_4_] isomorph derived using the Mercury code [45]. Therefore, given the identical peak positions and intensities, the pattern has been attributed to the [Gd_2_(BDC)_3_(H_2_O)_4_] phase. BDC ligands are coordinated to Gd^3+^ ions and together with the water molecules complete the eight coordination, the most common for gadolinium monomeric complexes [46,47], giving rise to the 3D network.

A further validation of the [Gd_2_(BDC)_3_(H_2_O)_4_] structure has been obtained by the Fourier transform infrared spectroscopy (FT-IR). The spectra, recorded in the 4000–500 cm^−1^ range (Figure S1), provide evidence of the presence of H_2_O molecules in the coordination sphere of the metallic center as assessed by the presence of a broad band around 3450 cm^−1^. Other significant peaks are observed at 1540, 1509, 1305 cm^−1^ and they are due to the stretching vibrations of the carboxylate C = O group. Peaks in the fingerprint region (1000–700 cm ^−1^) are due to the vibrational modes’ characteristic of the benzene ring. Peaks at 2923 cm^−1^, at 1460 cm ^−1^ and at 1377 cm^−1^ are due to the nujol used to prepare the mull.

**Figure 2:**
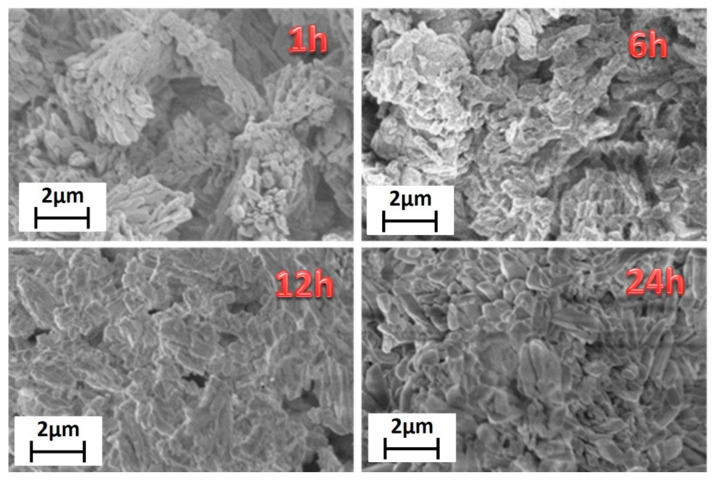
FE-SEM images of the [Gd_2_(BDC)_3_(H_2_O)_4_] synthesized for 1 h, 6 h, 12 h and 24 h.

Finally, morphological characterization has been carried out through field emission scanning electron microscopy (FE-SEM) using a very low acceleration voltage of 3 kV to avoid charging effects. FE-SEM images (Figure 2) show very homogeneous samples in terms of shape and dimensions.

Lower reaction time, 1 h, gives rise to bundles of nanorods of about 200–300 nm in diameter. Upon increasing the reaction time, the grain size increases, with dimensions of about 500–600 nm for the highest reaction time of 24 h. This is due to the effect of temperature for a prolonged time that allows a sintering of the network structure yielding larger and more crystalline grains. The energy dispersive X-ray (EDX) analysis (Figure S2) confirms the formation of the Gd-MOF structure, the spectrum showing all the expected peaks: the L and M emission lines of Gd are observed in the 6–7.5 keV range and at 1.2 keV, respectively; the C Kα and O Kα peaks found at 0.28 and 0.52 keV, respectively; the Au M peaks due to the sputtered layer around 2.1 keV.

### 3.3. Structural, Morphological and Compositional Properties of [Gd_2_(NDC)_3_(H_2_O)]·2(H_2_O)

**Figure 3:**
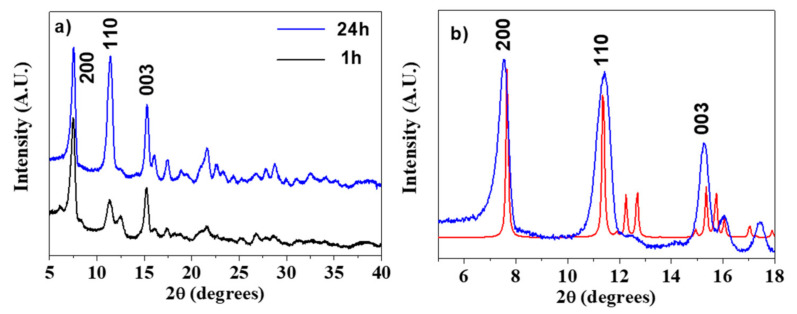
(**a**) X-ray powder patterns of the Gd-NDC networks synthesized for 1 and 24 h; (**b**) assignment of the [Gd_2_(NDC)_3_(H_2_O)]·2(H_2_O) pattern (blue line) in comparison with the Mercury derived powder pattern of the homologous [Yb_2_(NDC)_3_(H_2_O)]·2(H_2_O) (red line, ref. [48]).

Analogously to the Gd-BDC samples, the Gd-NDC with the 2,6-naphthalendicarboxylic acid was synthesized using water as a solvent and changing the reaction time, from 1 h to 24 h. In this case the reaction time has a slight effect on the crystallinity, as well. A comparison between the XRD patterns of Gd-NDC samples, obtained at 1 and 24 h, is reported in Figure 3a. The structure has been assigned by comparing the XRD pattern to the powder patterns of the homologous Ln-NDC [48,49] derived using the Mercury code [45]. In Figure 3b, the pattern of the Gd-NDC MOF (blue line) is perfectly coincident with that of the [Yb_2_(NDC)_3_(H_2_O)]·2(H_2_O) (red line) [48]. Therefore, the structural arrangement of the NDC ligand and H_2_O molecules gives rise to the [Gd_2_(NDC)_3_(H_2_O)]·2(H_2_O) network with a coordination of 6 or 7 around Gd.

The coordination number of 6 or 7 is lower than the most commonly observed 8-coordination for Gd monomeric complexes [46,47], but it is quite common for these systems where the presence of a ditopic linker gives rise to very complex networks.

A comment deserves the nature of water molecules found in the present Gd-NDC system, since there are both water molecules coordinated to the metal ions and free lattice water molecules.

**Figure 4:**
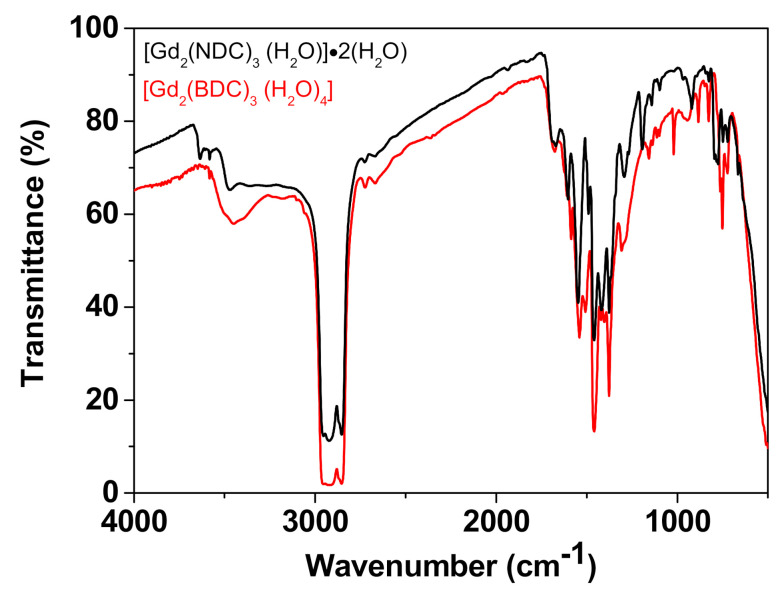
Comparison of the IR spectrum of the [Gd_2_(NDC)_3_(H_2_O)]·2(H_2_O) vs. the spectrum of the [Gd_2_(BDC)_3_(H_2_O)_4_].

This observation is further corroborated by the FT-IR spectra of the Gd-NDC, synthesized for 1, 6, 12, 24 h (Figure S3). These spectra provide evidence of the presence of two different kinds of very weak peaks in the range 3400 and 3700 cm^−1^: a broad small peak at 3470 cm^−1^ and sharp small peaks at 3582 and 3635 cm^−1^. The different kind of peaks may be associated, respectively, with the coordinated water molecules and the free lattice H_2_O molecules linked to the cavity cage through H-bonds [50,51,52]. It is worth to note the difference between the IR spectra in this region for the Gd-NDC vs. the Gd-BDC systems (Figure 4), that further confirms the dissimilar water role in the two networks. In fact, the [Gd_2_(BDC)_3_(H_2_O)_4_] spectrum shows only one broad peak in the 3400–3700 cm^−1^ region confirming the H_2_O coordination proposed for the Gd-BDC network, while coordinated and free lattice H_2_O molecules give rise to the different peaks found for the [Gd_2_(NDC)_3_(H_2_O)]·2(H_2_O) system.

Significant peaks are observed at 1603, 1540, 1416, 1295, 1119 cm^−1^ and are due to vibrations of the functional groups of the carboxylate. Other peaks found in the fingerprint region (1000–700 cm^−1^) can be attributed to the vibrational modes’ characteristic of the naphthalene ring.

**Figure 5:**
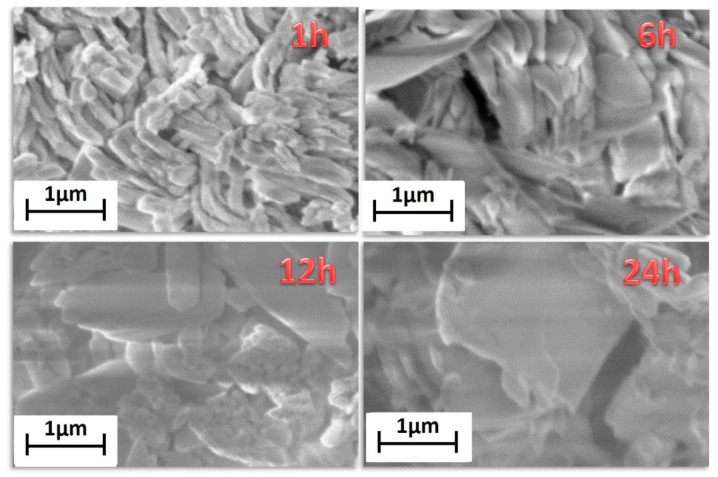
FE-SEM images of the [Gd_2_(NDC)_3_(H_2_O)]·2(H_2_O) synthesized for 1 h, 6 h, 12 h and 24 h.

The FE-SEM images (Figure 5) show homogeneous and well-structured samples. The sample synthesized for 1 h has rod-like grains of about 200 nm × 1000 nm. The increasing of the reaction time affects not only the crystalline structure, but also the morphology that changes from rod-like to plate-like grains. For the longer reaction time of 24 h the grains are almost equidimensional and of the order of micrometers.

### 3.4. Surface Area Measurements of Synthesized Gd-MOFs

The Brunauer–Emmett–Teller (BET) method has been applied to characterize the networks in terms of surface area, in order to evaluate the textured properties of MOFs for applications as heterogeneous catalysts or sensing materials.

In order to correlate the processing variables to the structure/surface area of Gd-MOFs, the [Gd_2_(BDC)_3_(H_2_O)_4_] synthesized at 1 h and 24 h have been analyzed to compare products obtained for short and long reaction times. The FE-SEM images (Figure 2) show grain dimensions of about 200–300 nm for the sample synthesized in 1 h, while for the 24 h treated sample the grains dimensions are in the range of 500–600 nm. Therefore, it can be inferred that longer reaction times affect the grain dimensions, also determining a smaller surface area, which is 70 ± 2 m^2^/g for the 24 h treated sample with respect to 83 ± 2 m^2^/g for the 1 h treated one.

In the case of [Gd_2_(NDC)_3_(H_2_O)]·2(H_2_O), the obtained data indicate that for the synthesis carried out in 1 h, the nanorod-like morphology (Figure 3) gives rise to a higher surface area of 75.2 ± 0.2 m^2^/g with respect to the 24 h synthesis, which produces a plate-like structures with larger dimensions and a consequent lower surface area 65 ± 2 m^2^/g.

In Table 1, surface areas and grain dimensions are compared, pointing to an effect of reaction time, which, in both cases, determine the formation of larger grains at 24 h and, consequently, smaller surface areas.

### 3.5. Sensing of Inorganic Ions

As a proof of concept, the properties of the [Gd_1.6_Eu_0.4_(BDC)_3_(H_2_O)_4_] and the [Gd_2_(BDC)_3_(H_2_O)_4_] systems, as fluorescent probes, have been tested for the detection of the most common metals and toxic anions contaminants such as Fe^3+^ and Cr^6+^, respectively. The 1 h synthesis has been chosen for the Gd,Eu-BDC MOF, since the Gd-BDC synthesized for 1 h presents the higher surface area of 83 ± 2 m^2^/g (see Table 1). Given the analogous ionic radii of Eu^3+^ and Gd^3+^, the Eu-doped Gd-BDC MOF is isostructural to the [Gd_2_(BDC)_3_(H_2_O)_4_], and thus it possesses a similar structure and characteristics.

A preliminary study of luminescence properties has been conducted through photoluminescence measurements in aqueous medium, using dispersions of the [Gd_2_(BDC)_3_(H_2_O)_4_], the [Gd_2_(NDC)_3_(H_2_O)]·(H_2_O)_2_ and the [Gd_1.6_Eu_0.4_(BDC)_3_(H_2_O)_4_], respectively (see Figure S4). Both the BDC-based MOFs show fluorescence emission at 265 nm excitation, while the Gd-NDC MOF has been excited at 285 nm. In particular, a broad emission occurs in the range of 400–450 nm for both the Gd-BDC and Gd,Eu-BDC systems and in the range of 350–400 nm for the Gd-NDC MOF, which may be attributed to a ligand-to-metal charge transfer [53]. The peak at 530 nm is a double-frequency scattering peak, whose wavelength is 2 times the excitation wavelength (265 nm). Moreover, the spectrum of the Gd,Eu-BDC MOF presents the characteristic emission of Eu^3+^ ion, with sharp and intense lines centered at 592 and 615 nm, which can be ascribed to the ^5^D_0_ → ^7^F_1_ and ^5^D_0_ → ^7^F_2_ transitions, respectively. Furthermore, under UV irradiation a strong red emission can be easily observed also by the naked eye (see inset in Figure S4).

**Figure 6:**
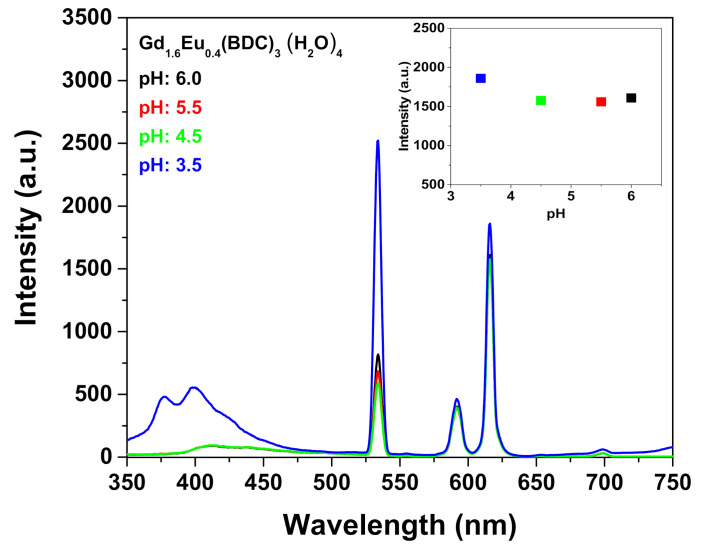
Emission spectra of the Gd_1.6_Eu_0.4_(BDC)_3_(H_2_O)_4_ (2 mg) dispersed into aqueous solution at different pH under excitation at 265 nm. The insert reports the intensity of the peak at 615 nm in the pH range 3.5–6.

Firstly, the use of [Gd_1.6_Eu_0.4_(BDC)_3_(H_2_O)_4_] MOF as sensing system has been tested for the detection of metal ions exploiting the excellent water stability and the high luminescent properties of the complex. Particularly, the as-prepared [Gd_1.6_Eu_0.4_(BDC)_3_(H_2_O)_4_] complex obtained at 1 h of synthesis has been dispersed into aqueous solution and different amounts of Fe^3+^ 1·× 10 ^−2^ M have been added to the suspension. Great care has been dedicated to have a homogeneous dispersion and to avoid any effect of sedimentation, thus the mixtures have been sonicated for 5 min after each addition to produce the metal ion-incorporated suspension for luminescent measurements. An incubation time of 5 min has been chosen to allow an optimal interaction between the MOF and the detected species. Particular attention has been devoted to the pH monitoring of the suspension during the sensing process to avoid instability of the detected species or the collapse of the MOF structure. This last phenomenon would be critical for the sensing process since the luminescence decrease would be related to the pH and not to the sensed species. The pH of the Eu-doped Gd-BDC system during the Fe^3+^ sensing detection varied from a 6.1 value at starting point (0 μL Fe^3+^ addition), to 4.2 at the end of the process (500 μL Fe^3+^ addition). Conversely, the pH remains almost unchanged, from 6.1 to 5.7, for the sensing of the Cr_2_O_7_^2−^. The pH variation for the Fe^3+^ sensing is due to the hydrolysis of Fe^3+^. To confirm that the luminescence decrease is only dependent on the ion detection, luminescence spectra of the [Gd_1.6_Eu_0.4_(BDC)_3_(H_2_O)_4_] have been recorded in the pH range 3.5–6.0 (Figure 6). The intensity of the Eu^3+^ peak at 615 nm is almost independent on pH (inset Figure 6) in the investigated pH range, but at the lowest pH of 3.5 an increase of the band in the range 350–420 nm is likely due to the MOF structure collapse and formation of the free ligand [27]. The observed pH dependence is in accordance with literature data [26,29].

**Figure 7:**
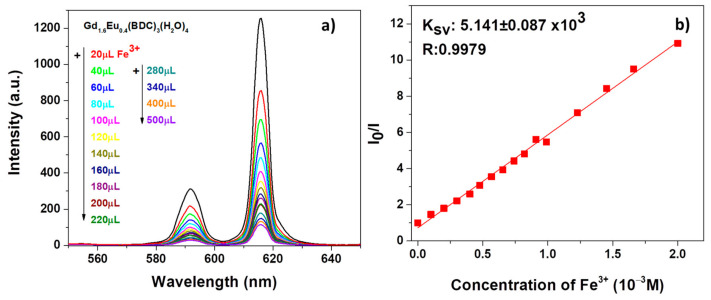
(**a**) Emission spectra and (**b**) Ksv curve Gd_1.6_Eu_0.4_(BDC)_3_(H_2_O)_4_ (2 mg) dispersed into aqueous solution in the presence of various concentrations of Fe^3+^ under excitation at 265 nm.

The result in Figure 7a reveals a strong quenching effect on the Eu^3+^ luminescent emission, already appreciable after the first addition of 20 μL of Fe^3+^ contaminant. The intensity of both the ^5^D_0_ → ^7^F_1_ and ^5^D_0_ → ^7^F_2_ decreases significantly upon increasing the Fe^3+^ amounts.

**Figure 8:**
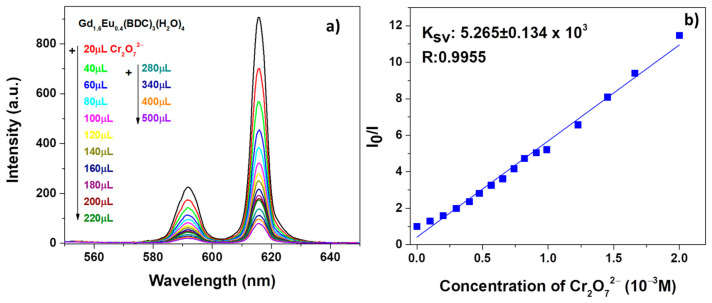
(**a**) Emission spectra and (**b**) Ksv curve Gd_1.6_Eu_0.4_ (BDC)_3_(H_2_O)_4_ (2 mg) dispersed into aqueous solution in the presence of various concentrations of Cr_2_O_7_^2−^ under excitation at 265 nm.

Similarly, promising sensing properties have been observed for the test on Cr_2_O_7_^2−^ solution as revealed in Figure 8a.

In order to have a quantitative estimation of the phenomena, the quenching effect has been rationalized by the Stern−Volmer equation [54] (see Figure 7b and Figure 8b):

I_0_/I = 1 + K_sv_[M] (3)

in which I_0_ and I are the intensity values of the most intense peaks (615 nm) of the Gd_1.6_Eu_0.4_(BDC)_3_(H_2_O)_4_ spectrum in the absence and presence of analytes, Ksv is the quenching constant and [M] is the analyte absolute concentration [55]. For both systems, in Figure 7b and Figure 8b, the linear coefficients R, very close to 1, point to linear correlations and so a good fit of the Stern−Volmer equation model. In addition, the quenching effect on the luminescence of Gd_1.6_Eu_0.4_(BDC)_3_(H_2_O)_4_ has been quantitatively estimated through the Ksv values, which have been calculated as 5.141 × 10^3^ and 5.265 × 10^3^ for Fe^3+^ and Cr_2_O_7_^2−^, respectively. The derived values of Ksv point to a strong quenching effect on the Eu^3+^ luminescence and thus a high sensitivity of the Eu-doped Gd-BDC MOF to ions detection. The presently found values are in accordance with literature data [56,57,58], and this represents a promising result considering that, in the present case, only an Eu-doped sample is used and thus the test would be a cost-effective sensing process, with respect to pure Eu-MOF structures.

Finally, to better understand the sensing phenomena, an accurate check of both framework structure and composition has been performed through XRD and EDX analysis on the Gd_1.6_Eu_0.4_(BDC)_3_(H_2_O)_4_ system before and after the Fe^3+^ sensing test. Three main causes may be responsible for the alteration of the MOF luminescence: (a) damage of the crystal structure; (b) cation exchange between the MOF ion and the sensed cations; and (c) physisorption interactions between the sensed species and the framework. The XRD pattern of Gd,Eu-BDC MOF after the detection of Fe^3+^ is identical to the untreated one (see Figure S5), thus proving that the structure is maintained. In addition, the EDX analysis supports this observation, since it does not reveal the presence of any Fe^3+^ contaminant within the detectability limit of the technique (Figure S6). These findings confirm that the framework structure remains unchanged after the test proving that the MOF framework is stable in the aqueous solution of Fe^3+^ ions and the fluorescence quenching phenomena is not due to the collapse of the crystal framework structures. The most likely prominent mechanism is the physisorption of Fe^3+^, which quenches the Eu luminescence [56]. Nevertheless, a comprehensive study on the nature of interaction would require much more time and investigation. Finally, MOFs’ size, after their applications in the ion sensing test, has been determined through FE-SEM images. Unfortunately, it is not possible to see disperse particles, but aggregates are visible (Figure S7). This could not be avoided since the samples have to be prepared by dropping the dispersion on an SEM Al stub and during water evaporation aggregation occurs.

These data point on one hand to assume that just a physisorption process occurs during the sensing test and, on the other hand, they confirm the high stability of the Gd_1.6_Eu_0.4_(BDC)_3_(H_2_O)_4_ system in water solution and after sensing applications.

In order to assess the reusable performance of the Gd,Eu-BDC MOF, sequential Fe^3+^ detection tests, using the same powder have been carried out repeatedly for three times (Figures S8 and S9). After each test the powder has been carefully washed, centrifuged and reused. The pH was monitored during the tests as well.

The pure Gd-BDC MOF has been also applied for the detection of the same ions, i.e., Fe^3+^ and Cr_2_O_7_^2−^, using identical procedures to those used for the tests with the Gd,Eu-BDC MOF. Results of the test done with Fe^3+^ ions (Figure S10a) indicate that the Gd-BDC MOF system does not respond to the Fe^3+^ detection, since a constant intensity of the broad emission between 400 and 550 nm is observed, as confirmed by the K_SV_ value (Figure S10b), whose error is higher than the value itself. This means that neither the band due to the ligand-to-metal charge transfer nor the specific band emission of Gd are sensitive to the Fe^3+^ detection. At difference, the Gd-BDC MOF is functional for the Cr_2_O_7_^2−^ sensing, since the emission of both bands decreases upon Cr_2_O_7_^2−^ addition (Figure S11a) and a K_SV_ value of 2.913 × 10^3^ is found (Figure S11b). Nevertheless, considering the low intensity of the Gd emission spectra with respect to the Gd,Eu-BDC MOF, the Eu doped system is overall more suited for ion detection.

## 4. Conclusions

The focus of this work is the development of a green, one-pot synthetic procedure for the preparation of Gd-MOFs and Gd,Eu-MOFs using the 1,4-benzenedicarboxylic acid or 2,6-naphthalenedicarboxylic acid as ditopic ligands. The syntheses were carried out from commercially available reagents, such as the Gd or Eu acetate salt, in H_2_O solvent and under mild operating conditions, ambient pressure and relatively short times from 1 h to 24 h. A systematic study has allowed to correlate the Gd-MOF textural properties to the synthetic processing conditions. The Gd-BDC and the Gd,Eu-BDC MOFs, synthesized in 1 h, have been applied to sensing inorganic ions, such as the Fe^3+^ and Cr_2_O_7_^2−^, in water solution yielding results comparable to materials obtained through the solvothermal process. Validation of applicability of the present system has been assessed through sequential ion detection tests, using the same powder for three times. These findings represent a great advantage if compared with data reported in the literature where the solvothermal synthesized MOFs, requiring days of reaction time and organic solvent such as dimethylformamide, are applied. In addition, the high stability in water of presently synthesized Ln-BDC MOFs accomplishes the mandatory requirement that they need to be stable in aqueous solution for real applications. These results indicate that the presently reported Eu-doped Gd MOF systems are suitable for inorganic ion detection and may be applied in various different sensing tests.

## Figures and Tables

**Table 1:** Surface area and grain dimensions of the 1 h and 24 h Gd-MOF samples with BDC and NDC linkers.

Sample	[Gd_2_(BDC)_3_(H_2_O)_4_]		[Gd_2_(NDC)_3_(H_2_O)]2(H_2_O)	
Reaction time	1 h	24 h	1 h	24 h
Surface area	83 ± 2 m^2^/g	70 ± 2 m^2^/g	75.2 ± 0.2 m^2^/g	65 ± 2 m^2^/g
Grain dimensions	200–300 nm	500–600 nm	300–350 nm	900–1000 nm

## Data Availability

The data presented in this study are available in the article and supplementary material.

## Supplementary Materials

The following are available online at https://www.mdpi.com/1424-8220/21/5/1679/s1, Figure S1: IR spectra of the Gd-BDC; Figure S2: EDX spectrum of the Gd-BDC; Figure S3: IR spectra of the Gd-NDC; Figure S4: Photoluminescence spectra of the Gd-BDC and Gd-NDC; Figure S5: XRD patterns of the Gd,Eu-BDC before and after Fe^3+^ sensing test; Figure S6: EDX spectra of the Gd,Eu-BDC before and after Fe^3+^ sensing test; Figures S7 and S8: Second and third run of Fe^3+^ detection using Gd,Eu-BDC; Figure S9: Fe^3+^ sensing test using the Gd-BDC; Figure S10: Cr_2_O_7_^2−^ sensing test using the Gd-BDC.
