# Supplementary material for: A Facile One-Pot Approach to the Synthesis of Gd-Eu Based Metal-Organic Frameworks and Applications to Sensing of Fe3+ and Cr2O72− Ions
Source: Sensors (Basel). 2021 Mar 1;21(5):1679. doi: 10.3390/s21051679 (PMC7957569; doi:10.3390/s21051679)
Supplement: Supplementary file 1 [file sensors-21-01679-s001.pdf]

sensors

Supplementary Materials

A Facile One-Pot Approach to the Synthesis of Gd-Eu Based Metal-Organic Frameworks and Applications to Sensing of Fe^{3+} and $Cr_2O_7^{2-}$ Ions

Roberta Puglisi [1,†], Anna L. Pellegrino [2,†], Roberto Fiorenza [1], Salvatore Scirè [1] and Graziella Malandrino [2,*]

[1] Dipartimento di Scienze Chimiche, Università degli Studi di Catania, Viale Andrea Doria 6, I-95125 Catania, Italy; robertapuglisi90@gmail.com (R.P.); rfiorenza@unict.it (R.F.); sscire@unict.it (S.S.)
[2] Dipartimento di Scienze Chimiche, Università degli Studi di Catania, INSTM UdR Catania, Viale Andrea Doria 6, I-95125 Catania, Italy; annalucia.pellegrino@unict.it
* Correspondence: graziella.malandrino@unict.it; Tel.: +39-095-7385055
† These authors have contributed equally to the study.

Citation: Puglisi, R.; Pellegrino, A.L.; Fiorenza, R.; Scirè, S.; Malandrino, G. A Facile One-Pot Approach to the Synthesis of Gd-Eu Based Metal-Organic Frameworks and Applications to Sensing of Fe^{3+} and $Cr_2O_7^{2-}$ Ions. *Sensors* **2021**, *21*, 1679. https://doi.org/10.3390/s21051679

Academic Editor: Georgia-Paraskevi Nikoleli

Received: 30 December 2020
Accepted: 24 February 2021
Published: 1 March 2021

Publisher's Note: MDPI stays neutral with regard to jurisdictional claims in published maps and institutional affiliations.

Copyright: © 2021 by the authors. Licensee MDPI, Basel, Switzerland. This article is an open access article distributed under the terms and conditions of the Creative Commons Attribution (CC BY) license (http://creativecommons.org/licenses/by/4.0/).

Gd-MOF characterization: The Gd-MOF have been characterized by Fourier transform infrared (FT-IR) in the 4000-500 cm^{-1} range.

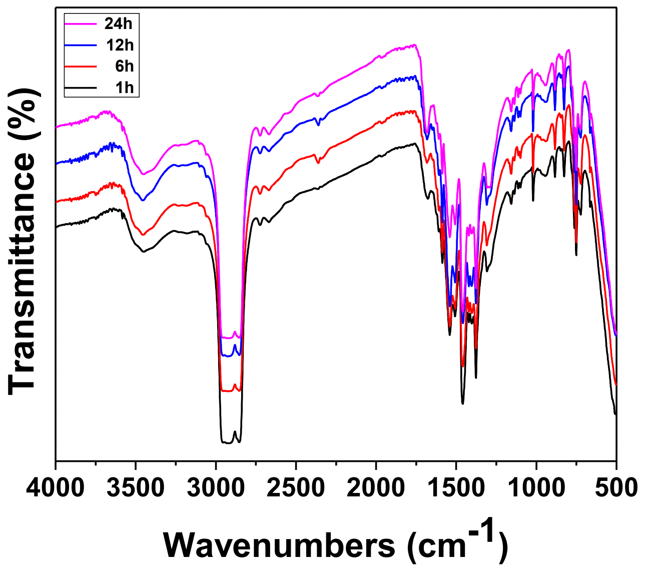

Figure S1. Comparison of IR spectra of the $Gd_2(BDC)_3(H_2O)_4$ complexes synthesized for 1, 6, 12 and 24 h.

The energy dispersive X-ray (EDX) analysis of the sample obtained in a 6 hours synthesis is reported to verify the composition of the MOF.

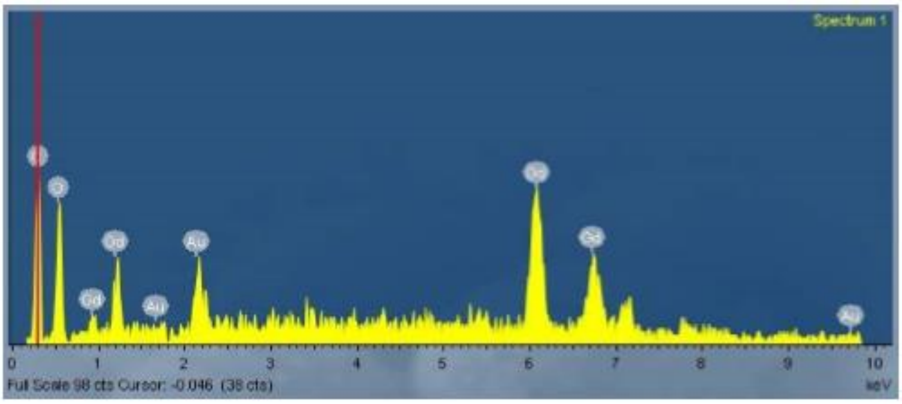

Figure S2. EDX spectrum of the $[Gd_2(BDC)_3(H_2O)_4]$ complex synthesized for 6h.

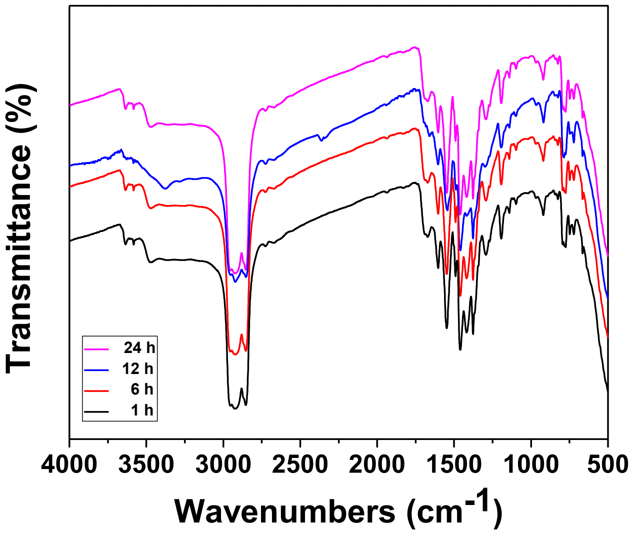

Figure S3. Comparison of IR spectra of the $[Gd_2(NDC)_3(H_2O)]\cdot(H_2O)_2$ complexes synthesized for 1, 6, 12 and 24 h.

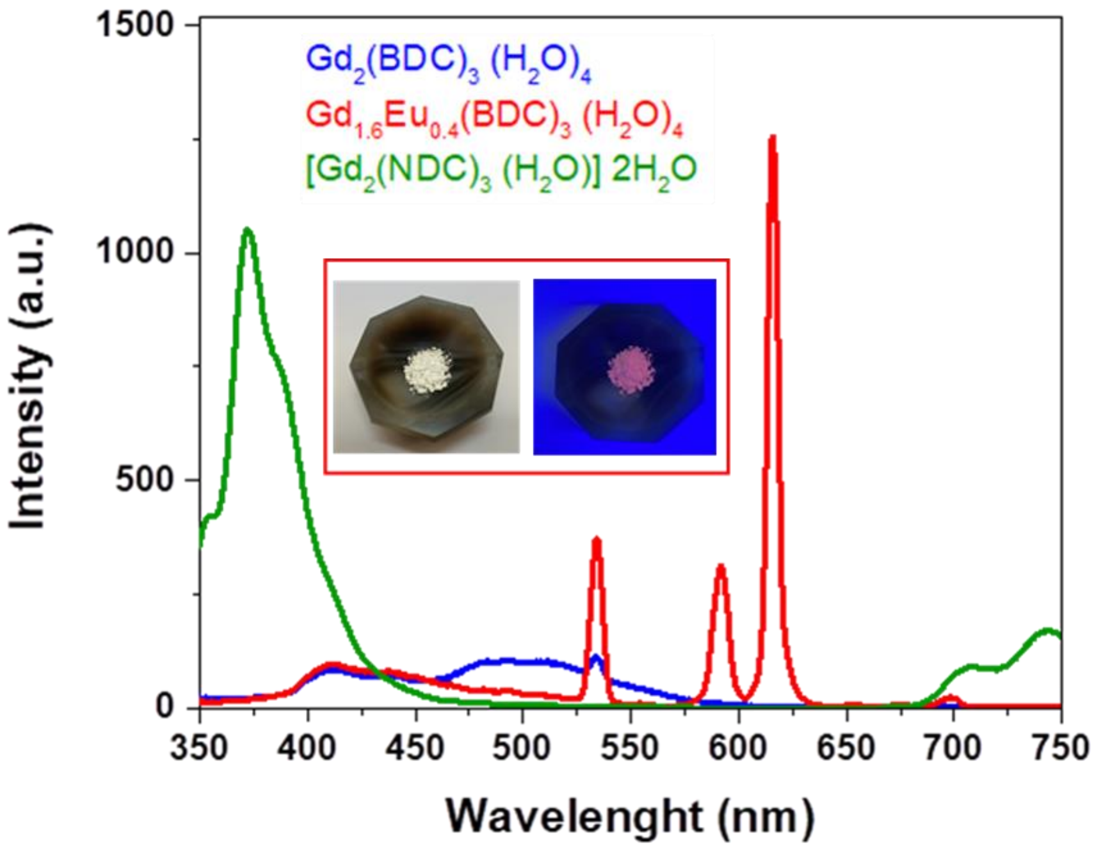

Figure S4. Photoluminescence spectra of aqueous dispersions of the [$Gd_2(BDC)_3(H_2O)_4$] (blue line), the [$Gd_2(NDC)_3(H_2O)$]·$(H_2O)_2$ (green line) and the [$Gd_{1.6}Eu_{0.4}(BDC)_3(H_2O)_4$] (red line) obtained at 1h (λ ecc: 265 nm for the BDC based MOFs and 285 nm for the Gd-NDC).

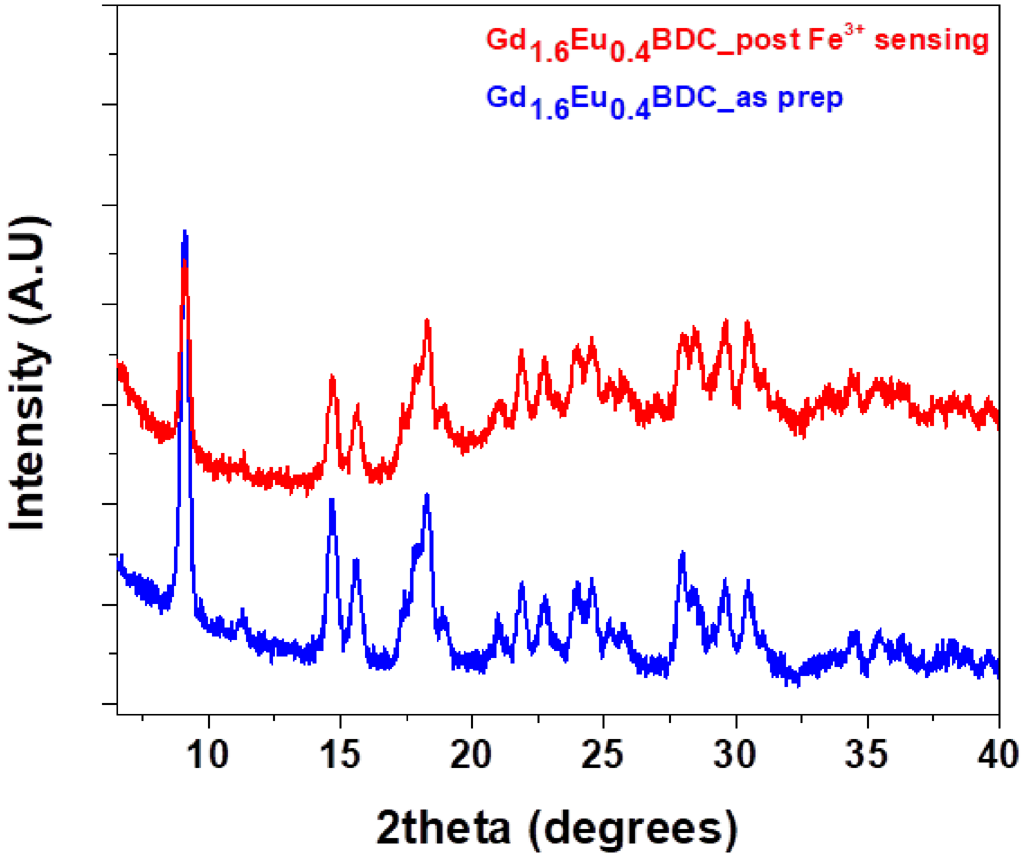

Figure S5. Comparison of the X-ray powder patterns of the $[Gd_{1.6}Eu_{0.4}(BDC)_3(H_2O)_4]$ complex synthesized for 1h before and after Fe^{3+} sensing test.

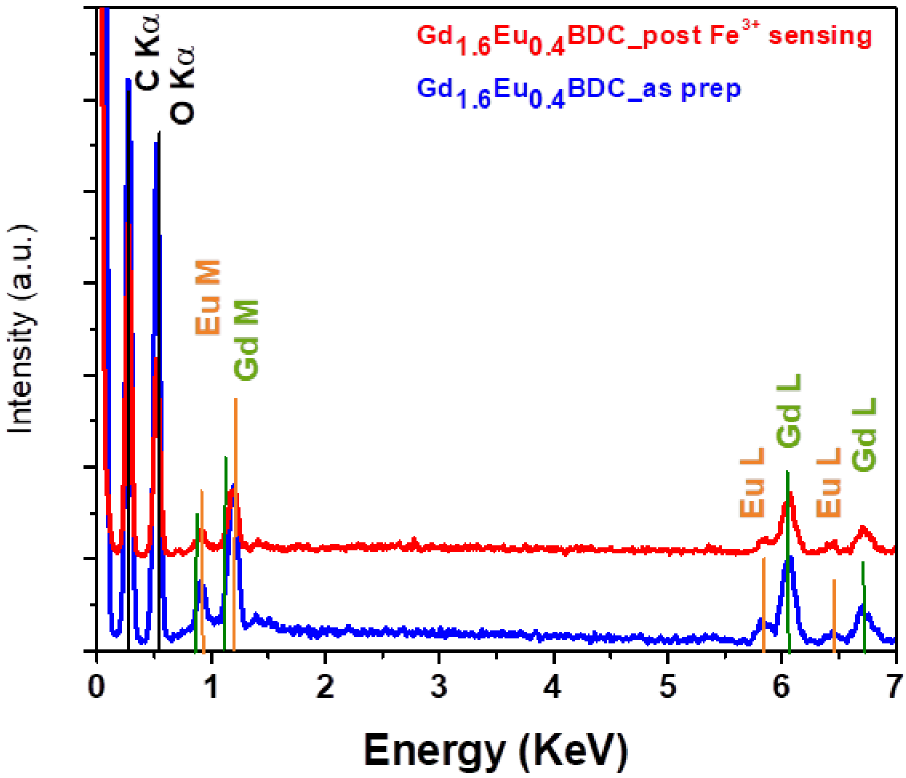

Sample	Quantitative analysis			
	C (K)	O (K)	Eu (L)	Gd (L)
As prep	63.09%	31.56%	0.98%	4.37%
Post Fe^{3+} sensing	69.86%	23.36%	1.04%	5.15%

Figure S6. EDX spectra and quantitative analysis of [$Gd_{1.6}Eu_{0.4}(BDC)_3\cdot(H_2O)_4$] complexes synthesized for 1h before and after Fe^{3+}sensing test.

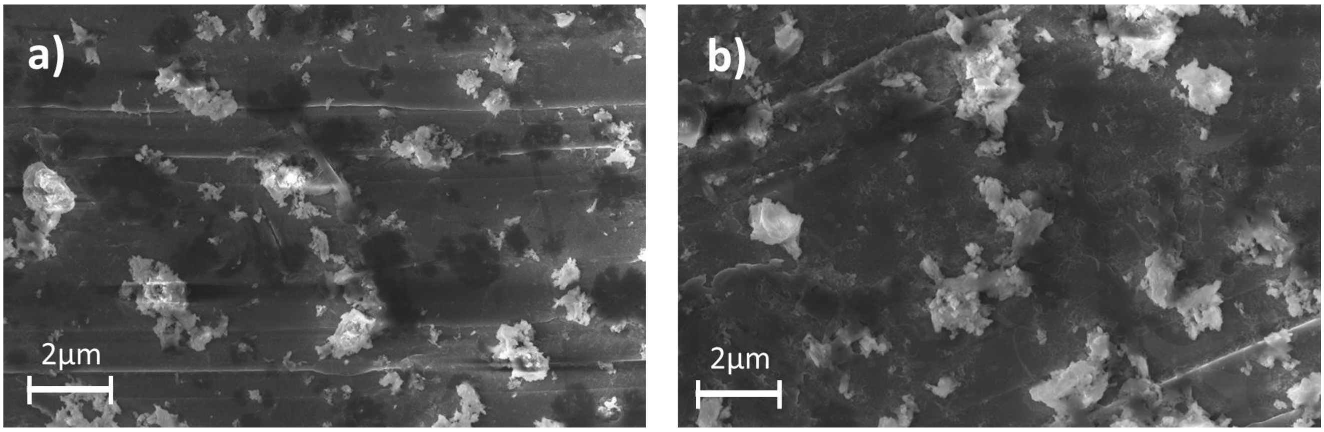

Figure S7. FE-SEM images of (**a**) the [$Gd_2(BDC)_3(H_2O)_4$] and (**b**) the [$Gd_{1.6}Eu_{0.4}(BDC)_3(H_2O)_4$] as aggregate distribution after suspension processes.

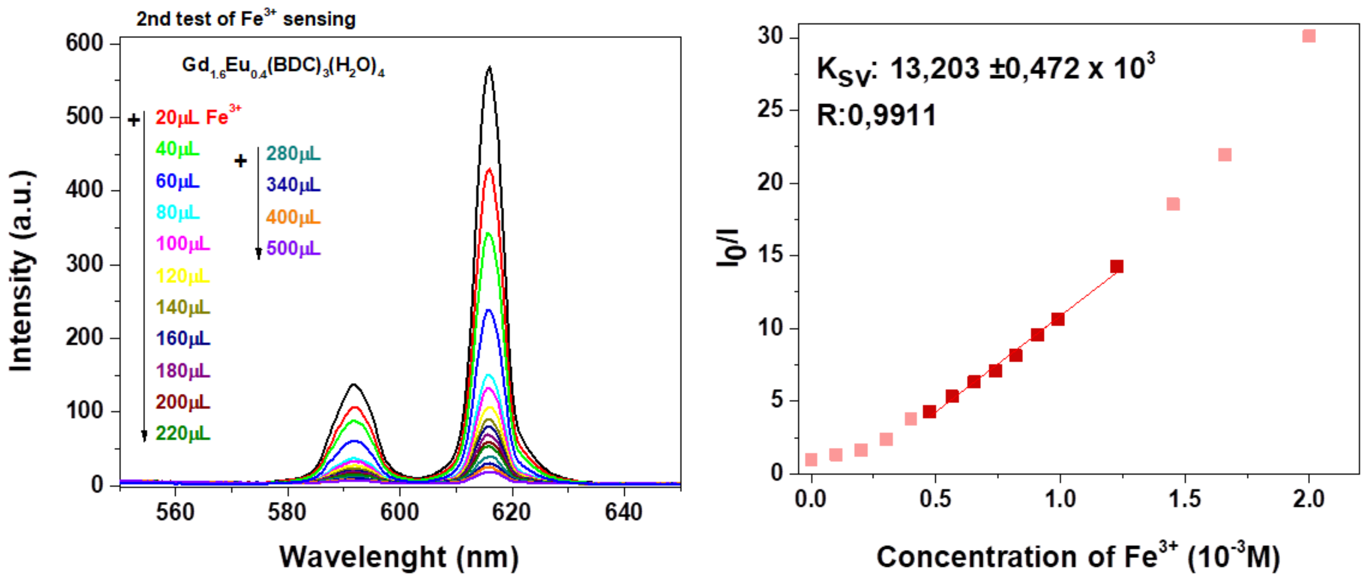

(**a**) (**b**)

Figure S8. (**a**) Emission spectra and (**b**) Ksv curve of second set of sensing test on $Gd_{1.6}Eu_{0.4}(BDC)_3(H_2O)_3$ (2 mg) dispersed into aqueous solution in the presence of various concentrations of Fe^{3+} under excitation at 265 nm.

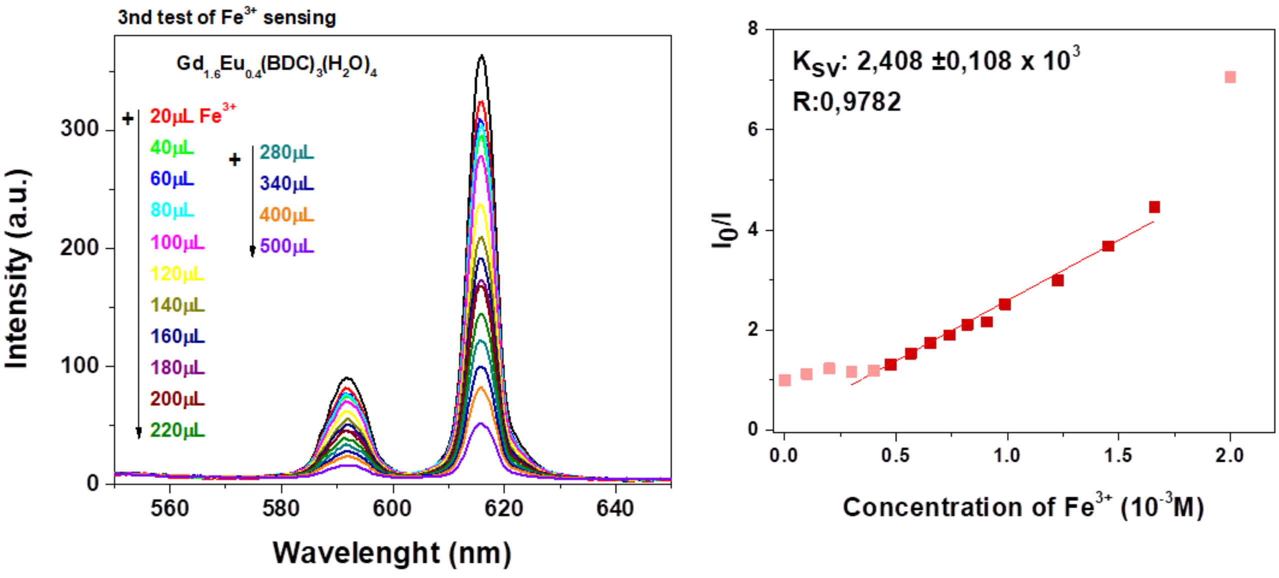

(**a**) (**b**)

Figure S9. (**a**) Emission spectra and (**b**) Ksv curve of third set of sensing test on $Gd_{1.6}Eu_{0.4}(BDC)_3(H_2O)_3$ (2 mg) dispersed into aqueous solution in the presence of various concentrations of Fe^{3+} under excitation at 265 nm.

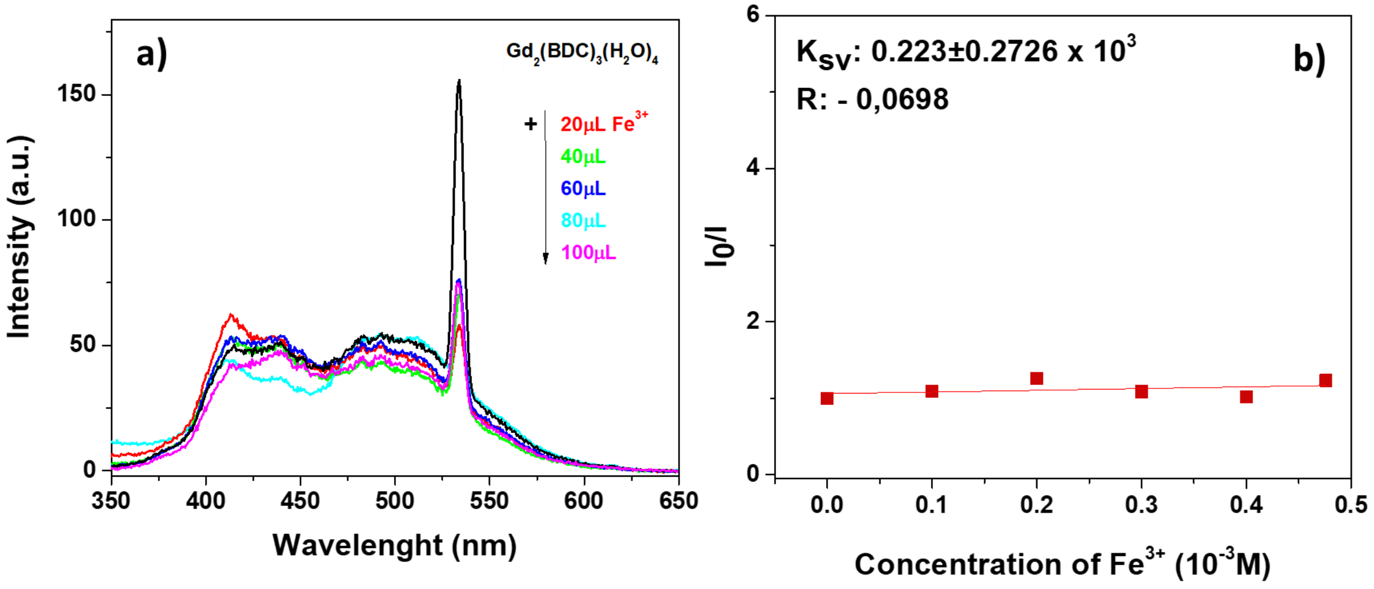

Figure S10. (**a**) Emission spectra and (**b**) Ksv curve of sensing test on $Gd_2(BDC)_3(H_2O)_3$ (2 mg) dispersed into aqueous solution in the presence of various concentrations of Fe^{3+} under excitation at 265 nm.

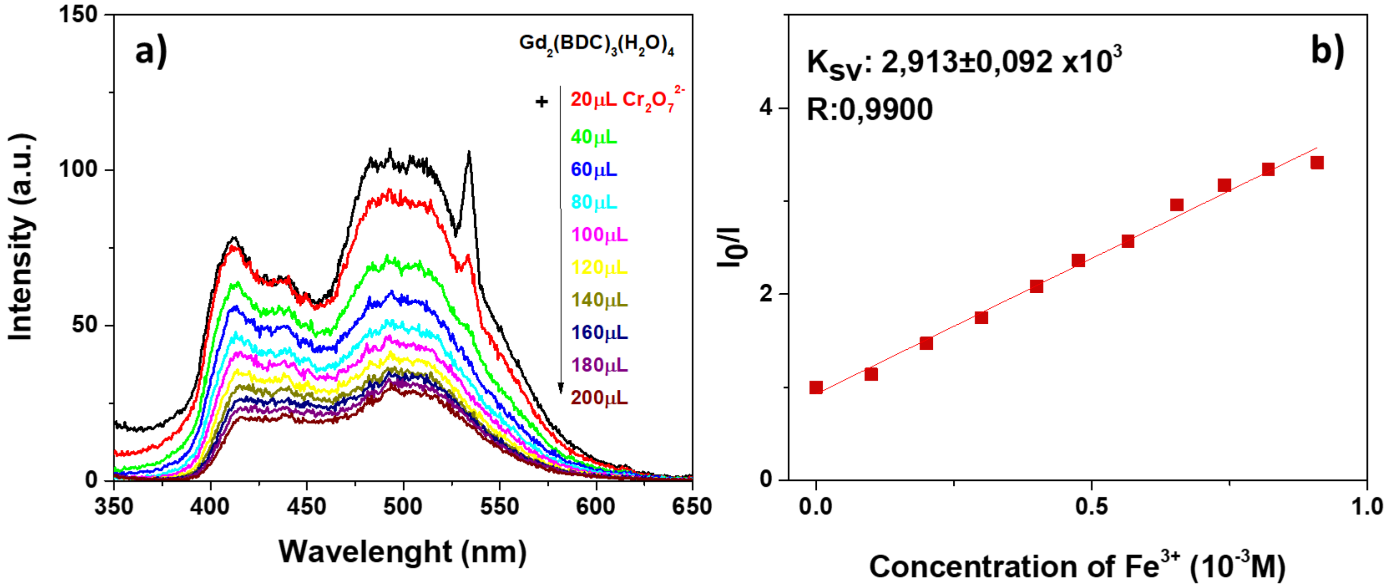

Figure S11. (**a**) Emission spectra and (**b**) Ksv curve of sensing test on $Gd_2(BDC)_3(H_2O)_3$ (2 mg) dispersed into aqueous solution in the presence of various concentrations of $Cr_2O_7^{2-}$ under excitation at 265 nm.
